# Lacrimal Gland Repair Using Progenitor Cells

**DOI:** 10.5966/sctm.2016-0191

**Published:** 2016-08-15

**Authors:** Anastasia Gromova, Dmitry A. Voronov, Miya Yoshida, Suharika Thotakura, Robyn Meech, Darlene A. Dartt, Helen P. Makarenkova

**Affiliations:** ^1^ Department of Cell and Molecular Biology, The Scripps Research Institute, La Jolla, California, USA; ^2^ Biomedical Sciences Graduate Program, University of California San Diego, La Jolla, California, USA; ^3^ Institute for Information Transmission Problems, Russian Academy of Sciences and A.N. Belozersky Institute of Physico‐Chemical Biology of the Lomonosov Moscow State University, Moscow, Russia; ^4^ Department of Clinical Pharmacology, Flinders University, Bedford Park, South Australia, Australia; ^5^ Department of Ophthalmology Harvard Medical School, Schepens Eye Research Institute/Massachusetts Eye and Ear Infirmary, Boston, Massachusetts, USA

**Keywords:** Cell culture, Cell transplantation, Cellular therapy, Tissue‐specific stem cells, Fluorescence‐activated cell sorting, c‐kit, Cell surface markers, Gene expression

## Abstract

In humans, the lacrimal gland (LG) is the primary contributor to the aqueous layer of the tear film. Production of tears in insufficient quantity or of inadequate quality may lead to aqueous‐deficiency dry eye (ADDE). Currently there is no cure for ADDE. The development of strategies to reliably isolate LG stem/progenitor cells from the LG tissue brings great promise for the design of cell replacement therapies for patients with ADDE. We analyzed the therapeutic potential of epithelial progenitor cells (EPCPs) isolated from adult wild‐type mouse LGs by transplanting them into the LGs of *TSP* *‐1^−/−^* mice, which represent a novel mouse model for ADDE. *TSP‐1^−/−^* mice are normal at birth but progressively develop a chronic form of ocular surface disease, characterized by deterioration, inflammation, and secretory dysfunction of the lacrimal gland. Our study shows that, among c‐kit‐positive epithelial cell adhesion molecule (EpCAM^+^) populations sorted from mouse LGs, the c‐kit^+^dim/EpCAM^+^/Sca1*^−^*/CD34*^−^*/CD45*^−^* cells have the hallmarks of an epithelial cell progenitor population. Isolated EPCPs express pluripotency factors and markers of the epithelial cell lineage Runx1 and EpCAM, and they form acini and ducts when grown in reaggregated three‐dimensional cultures. Moreover, when transplanted into injured or “diseased” LGs, they engraft into acinar and ductal compartments. EPCP‐injected *TSP‐1^−/−^* LGs showed reduction of cell infiltration, differentiation of the donor EPCPs within secretory acini, and substantial improvement in LG structural integrity and function. This study provides the first evidence for the effective use of adult EPCP cell transplantation to rescue LG dysfunction in a model system. Stem Cells Translational Medicine *2017;6:88–98*

**Significance Statement**

In humans, the lacrimal gland is the primary contributor to the aqueous layer of the tear film. Damage or inflammation of the lacrimal gland may lead to severe aqueous‐deficiency dry eye and corneal disease. Endogenous lacrimal gland epithelial cell progenitors (EPCPs) injected into the gland of mouse model of human Sjögren's syndrome *TSP‐1^−/−^* mice resulted in long‐term engraftment and markedly improved structure and function of “diseased” lacrimal gland. This study demonstrates, for the first time, that EPCPs can mediate functional recovery of the lacrimal gland in a Sjögren's syndrome mouse model. These data establish proof of concept that endogenous stem/progenitor cell transplantation may be used to treat human lacrimal gland chronic inflammation.

## Introduction

Aqueous‐deficiency dry eye (ADDE) is characterized by a lack of tear secretion from the lacrimal glands (LGs). ADDE affects millions of Americans, causing a debilitating loss of visual acuity, ocular surface irritation, and adverse lifestyle changes. In humans, the LGs are the primary contributor to the aqueous layer of the tear film, and many cases of ADDE, classified as aqueous surface dry eye, involve LG dysfunction and/or degeneration. One of the challenges of understanding the mechanism of human dry eye pathogenesis is the inability to perform biological and molecular studies before obvious clinical signs. As a result, the precise steps of disease development are not well understood. Currently there is no cure for advance cases of dry eye. Developing new therapies to restore LG function would drastically improve the quality of life of patients affected by ADDE. One possible new treatment option for ADDE is the use of stem/progenitor cells to induce LG regeneration. In many tissues (lung, muscle, brain, and heart), stem/progenitor cell‐based therapies have been demonstrated to be viable approaches to treating diseases previously considered incurable 1 2 3. Similar to other exocrine glands (pancreas, salivary, and mammary) 4 5 6 7, the “healthy” adult LG is highly regenerative and is able to repair itself, even after substantial damage 8, 9. For example, a single injection of interleukin‐1α (IL‐1α) induces a severe inflammatory response, leading to destruction of LG acinar and epithelial cells, followed by epithelial cell proliferation and complete LG regeneration. In contrast, “diseased” chronically inflamed LGs that also show structural damage/destruction do not effectively repair 10. The reason for this failure to repair is unclear, but may relate to chronic disruption of LG stem cell niche functions that are important to support stem cell‐mediated regeneration. There is evidence that the adult LG epithelium contains both slow‐cycling stem cells 11 and faster‐cycling progenitor cells 12, 13; however, the roles of these cells in LG regeneration remain undefined.

Recently, replacement of an adult mouse LG with an embryonic LG‐derived epithelio‐mesenchymal reaggregate has been demonstrated 9. However, obtaining human embryonic LGs would be a challenge, and they may not contain enough cells for adult LG restoration.

In this study, we report the isolation and characterization of putative epithelial cell progenitors (EPCPs) from adult uninjured LGs. These cells expressed c‐kit and markers of the epithelial cell lineage Runt‐related transcription factor 1 (Runx1) and epithelial cell adhesion molecule (EpCAM). When grown in reaggregated three‐dimensional (3D) cultures, EPCPs were able to differentiate into organoids containing multiple LG cell types. Moreover, when transplanted into injured LGs, EPCPs were able to engraft into acinar and ductal compartments.

We also tested the engraftment and function of EPCPs in the “diseased” LG using a recently reported model of ADDE—the thrombospondin‐1 null *TSP‐1^−/−^* (also known as *Thbs‐1^−/−^*) mouse. This model fully mimics the development of chronic autoimmune dry eye disease with loss of function preceding inflammatory cell infiltration 14. Thrombospondin‐1 is a component of the extracellular matrix that functions in cell‐matrix and cell‐cell interactions, regulation of cellular phenotype, and activation of latent transforming growth factor‐β 15, 16. Although born with no apparent abnormalities of the LG anterior segment, *TSP‐1^−/−^* mice develop a severe inflammation of the LG with inflammatory infiltrates containing CD4 and CD8 T cells. Similar to Sjögren's syndrome (SjS) patients, both anti‐SSA/Ro and anti‐SSB/La autoantibodies are detected at elevated levels in *TSP‐1^−/−^* mice sera, and a significant loss of LG secretory function is observed by 24 weeks 14. Elevated levels of interleukins 17 and 6 and interferon γ (IFN‐γ) in the LG of 8‐week‐old *TSP‐1^−/−^* mice are also observed.

Transplantation of EPCPs into a chronically inflamed LG of the *TSP‐1^−/−^* mouse restored the structural compartment of the “diseased” LG and increased tear secretion. This study provides new insight into LG progenitor cells that might be used in cell replacement therapy for degenerative dry eye diseases that still have no effective treatment.

## Materials and Methods

### Mice

*C57BL/6* (3–4 weeks old) mice were used for LG EPCP isolation and characterization. Donor and recipient mice were maintained in The Scripps Research Institute (TSRI) vivarium under standard conditions. Donor cells were isolated from *Rosa26‐LacZ (R26‐LacZ)* or *Pax6‐LacZ* mice. *R26‐LacZ* mice were generated by crossing *R26‐Cre* and *R26‐LacZ^flox/flox^* mice as described previously 17. The *Pax6‐LacZ* mouse was reported previously 18. Mice used as recipients for EPCP transplantations were *C57BL/6J* (wild‐type) and *TSP‐1^−/−^* mice that are a model of chronic LG inflammation/destruction. *TSP‐1^−/−^* mice were originally purchased from the Jackson Laboratory (Sacramento, CA, https://www.jax.org) and were bred and maintained on the *C57BL/6J* background at the Scripps Research Institute vivarium. All experiments were conducted in accordance with the Association for Research in Vision and Ophthalmology Statement for the Use of Animals in Ophthalmic and Vision Research and were approved by The Scripps Research Institute Animal Care and Use Committees.

### Culture Media

#### Dissection Medium

Eagle's Minimum Essential medium (MEM; catalog no. 12492‐013, Thermo Fisher Scientific Life Sciences, Oakwood Village, OH, https://www.thermofisher.com) without glutamine was supplemented with the Glutamax at 5 ml (catalog no. 35050‐061, Thermo Fisher), 10% fetal bovine serum (FBS), and antibiotic‐antimycotic (catalog no. 15240‐062, Thermo Fisher).

#### Growth Medium

EpiLife (Thermo Fisher) was supplemented with Human Corneal Growth Supplement (catalog no. S‐009‐5, Thermo Fisher), 5 ng/ml basic fibroblast growth factor (bFGF) (FGF2), 10 ng/ml epidermal growth factor (EGF) (R&D Systems Inc., Minneapolis, MN, https://www.rndsystems.com), and 10% FBS.

#### Differentiation Medium

Fitton‐Jackson Modification (BGJb) was supplemented with GlutaMAX, 0.1% Lipid‐Rich Bovine Serum Albumin (AlbuMAX I, catalog no. 11020, Thermo Fisher), insulin‐transferrin‐selenium (catalog no. 41400‐045, Thermo Fisher), human transferrin (catalog no. 0030124SA, Thermo Fisher), and 5 ng/ml EGF (R&D Systems).

### Immunostaining and Confocal Microscopy

LG sections, monolayer, and 3D cultures were stained by using an indirect sequential immunoenzymatic technique. The following primary antibodies were used for immunostaining: rabbit polyclonal antibody to Pax6 (PRB‐278P, Covance, Princeton, NJ, http://www.covance.com), mouse monoclonal E‐cadherin antibody (clone 36/E‐cadherin; catalog no. 610181, BD Biosciences, San Jose, CA, http://www.bdbiosciences.com; or clone DECMA‐1; EMD Millipore, Billerica, MA, http://www.emdmillipore.com), rat monoclonal antibody against Ser‐28 phosphohistone‐H3 (P‐H3) (clone HTA28; catalog no. H9908, Sigma‐Aldrich, St. Louis, MO, http://www.sigmaaldrich.com), laminin antibody produced in rabbit (catalog no. L 9393; Sigma‐Aldrich), and cytokeratin 5 (Krt5) rabbit polyclonal antibody (catalog no. AF138; Covance), mouse monoclonal α‐smooth muscle actin antibody (clone 1A4; catalog no. A2547, Sigma‐Aldrich), rat monoclonal antibody to platelet endothelial cell adhesion molecule (CD31) (clone MEC 13.3; catalog no. 557355, BD Biosciences), goat polyclonal antibody to thrombospondin‐1 (c‐20; catalog no. sc‐7653; Santa Cruz Biotechnology Inc., Santa Cruz, CA, http://www.scbt.com). Complimentary secondary antibodies were obtained from Thermo Fisher. Images were taken by using a Zeiss LSM 780 laser scanning confocal microscope (Zeiss, Stuttgart, Germany, http://www.zeiss.com).

### Western Blotting Analysis

Cultured undifferentiated and differentiated EPCPs were homogenized in radioimmunoprecipitation assay (RIPA) buffer and sonicated. Equal aliquots of protein were resolved by SDS‐polyacrylamide gel electrophoresis, transferred to polyvinylidene difluoride membrane, and probed with antibodies to Aquaporin‐5 (Aqp5; ab104751, Abcam, Cambridge, MA, http://www.abcam.com) and reprobed with the β‐actin antibody (clone AC‐15, A5441, Sigma‐Aldrich) (loading control), which acted as a reference standard. The bands were quantified by densitometry, and the ratio of Aqp5/β‐actin was calculated. Three experiments were performed, and the results were normalized to the values from spontaneously differentiated EPCP cultures (48 hours in the differentiation culture medium).

### Lacrimal Gland Cell Dissociation (Adult Mouse Uninjured Glands)

To obtain sufficient cells for flow cytometric analysis and fluorescence‐activated cell sorting (FACS), we pooled LGs from 6–12 mice. Mice were euthanized, and the skin was sterilized with 70% ethanol before surgically exposing the LG. The LG was dissected away from the closely associated parotid salivary gland and placed into phosphate‐buffered saline (PBS), pH 7.4. The LG capsule was removed with tweezers, and pooled LGs were treated with trypsin‐pancreatin solution 19 for 25–40 minutes at room temperature to promote penetration of collagenase at the next step. LGs were rinsed with dissection medium (MEM supplemented with 10% FBS and antibiotic‐antimycotic; catalog no. 15240‐062, Thermo Fisher) and minced in 2 ml of 2 mg/ml collagenase (type I, Thermo Fisher Scientific Life Sciences catalog no. 17100‐017) in dissection medium. LG fragments were incubated in a CO_2_ incubator for 30–60 minutes with gentle triturating to dissociate cells (timing varied by age group). Dissociated cells were filtered through a 70‐µm mesh cell strainer (catalog no. 352350, Corning Life Sciences DL, Kaiserslautern, Germany, https://www.corning.com), washed in dissection medium, resuspended, and refiltered. Cells were treated for 15 minutes in a solution of 100 µg/ml DNase I (Sigma‐Aldrich, catalog no. D‐4513) and 5 mM MgCl_2_ in Hanks’ balanced saline solution (HBSS) at room temperature. Finally, the isolated LG cells were resuspended in 5 ml of growth medium.

### Antibody Staining and Fluorescence‐Activated Cell Sorting

To remove red blood cells, 25 ml of cold red blood cell lysis buffer (0.2% [wt/vol] Tris pH 7.5, 0.747% [wt/vol] NH_4_Cl) was added to each tube of LG cells suspended in growth medium. Purified LG cells were collected by centrifugation at 1,000*g*, resuspended in 100 µg/ml DNase, 5 mM MgCl_2_ in HBSS (DNase, Sigma‐Aldrich D‐4513; HBSS, Sigma H‐6648) and incubated for 15–30 minutes at room temperature. The cells were pelleted at 1,000*g*, washed in HBSS, resuspended in staining buffer PBE (1× PBS, 0.5% bovine serum albumin, 1 mM EDTA) and counted. For FACS analysis, approximately 0.5 × 10^6^ cells were pelleted at 1,200*g* for 10 minutes at 4°C and resuspended in 100 μl of staining buffer with the appropriate conjugated antibody. The following antibodies were used: phycoerythrin (PE) rat anti‐mouse Ly‐6A/E (Sca1) (catalog no. 553336, BD Biosciences); PE‐Cy7 rat anti‐mouse CD31 (catalog no. 561410, BD Biosciences); FITC rat anti‐mouse CD34 (catalog no. 553733, BD Biosciences); anti‐mouse CD117 (c‐Kit) allophycocyanin (APC)‐eFluor 780 (catalog no. 47‐1171‐80, eBioscience, San Diego, CA, http://www.ebioscience.com); and anti‐mouse CD326 (EpCAM) APC (catalog no. 17‐5791‐80, eBioscience). The cells were stained on ice for 1 hour with gentle vortexing every 15–20 minutes, pelleted as above, and resuspended in 1 ml of cold PBE in FACS tubes.

Flow cytometric analysis and FACS were performed at the TSRI Flow Cytometry Core Facility by using Digital LSRII and FACS Vantage DiVa instruments. Data analyses were performed by using FlowJo software. The main population of cells was determined by forward and side scatter area gating, as well as dead cell exclusion via propidium iodide or 7‐aminoactinomycin D. Doublets were excluded via forward scatter area versus width and with side scatter area versus width ( supplemental online Fig. 1A, 1B). Control samples labeled with isotype control antibodies and with a single primary antibody were used to determine the background noise because of nonspecific antibody binding and to establish proper compensation for optimum separation between signals.

### Preparation of 3D Reaggregated EPCP Cultures

EPCPs were isolated by FACS as described above. Isolated EPCPs were counted and dissociated in culture medium. A total of 1 × 10^5^ to 1.5 × 10^6^ cells in 50 µl of medium were drawn into a 100‐µl pipette tip, and the tip was sealed with sterile parafilm. Cells were pelleted at 1,000*g* for 10 minutes and the cell plug/reaggregate then was inoculated into a 15‐µl drop of laminin‐111 (Trevigen, Gaithersburg, MD, https://www.trevigen.com) gel or Matrigel (BD Bioscience) resting on a polycarbonate filter and floating in serum‐free medium ( supplemental online Fig. 2).

### Reverse‐Transcriptase Profiler Polymerase Chain Reaction Array

Gene expression in sorted EPCPs (c‐kit^+^dim/EpCAM^+^ cells) were compared with isolated c‐kit^−^/EpCAM^+^ epithelial cells. RNA was isolated by using a RNeasy Mini kit (catalog no. 74104; SABiosciences, Qiagen, Valencia, CA, http://www.qiagen.com); cDNA was made by using an RT^2^ First Strand Kit (catalog no. 330401; SABiosciences, Qiagen) and applied to the Mouse Stem Cell Transcription Factors RT^2^ Profiler Array (Qiagen, PAMM‐501Z). Quantitative reverse‐transcriptase polymerase chain reaction was performed on the ABI 7300 system (Thermo Fisher), and data were analyzed by using online normalization and analysis tools (provided in the public domain, http://sabiosciences.com/pcrarraydataanalysis.php). Three independent experiments were performed.

### In Vitro Colony‐Forming Efficiency Assay

Colony‐forming efficiency (CFE) assays were performed by using FACS‐isolated EPCPs and c‐kit^−^/EpCAM^+^ epithelial cells (control) or EPCPs that were maintained in either monolayer or 3D cultures for 3 weeks. Monolayer and 3D cultures were trypsinized to prepare a single cell solution before CFE assays. All cells were washed, filtered, counted, and centrifuged in 15‐ml tubes at 300*g* for 10 minutes. The supernatant was removed, and cells were resuspended in an appropriate volume of culture medium by gentle pipetting to generate a single cell suspension as confirmed by microscopy. Cells were seeded at 1 × 10^3^ cells per square centimeter into coated six‐well dishes. The colonies were fixed at day 8 and stained with 10% methylene blue in 70% ethanol. The CFE was calculated as the number of colonies at day 8 as a proportion of the number of cells plated in a well. Colonies were manually scored under a stereomicroscope. The standard error of the mean was calculated for at least three independent experiments and at least three replicates for each experimental point.

### Tear Production Measurement

Tear production was measured by using phenol red‐impregnated cotton threads (Zone‐Quick; Lacrimedics, DuPont, WA, http://www.lacrimedics.com), as described previously 10, 20. The threads were applied to the ocular surface in the lateral canthus for 10 seconds. Wetting of the thread (which turns red in contact with tears) was measured in millimeters. Tear volume was measured in both eyes of recipient mice before and after progenitor cell transplantations. Only females with reduced tear production (2.5 ± 0.5 mm) were used in transplantation experiments.

### EPCP Cell Transplantation

Sorted EPCP cells were pelleted and resuspended in Minimum Essential Medium α (α‐MEM) (Corning). To analyze EPCP engraftment, we performed EPCP transplantation into injured LGs. LGs of 3‐ to 4‐month‐old WT recipient mice were injured by a single IL‐1 injection as described previously 10. Donor EPCP cells were obtained by cell sorting from uninjured LacZ^+^ LGs (see above) of 3‐ to 4‐month‐old WT mice and were transplanted into the recipient LGs 1 or 3 days after the injury. Approximately 4,000–5,000 cells in 3 μl of α‐MEM were slowly injected into the LG of anesthetized mice by using a 28‐G needle and a Microliter Syringe (Hamilton, Reno, NV, http://www.hamiltoncompany.com). Control LGs were injected with vehicle (α‐MEM). Transplantation was repeated for a total of three independent experiments. Analysis of EPCP engraftment was carried out 40 days after transplantation. To analyze engraftment and function of EPCPs in the chronically inflamed LG of *TSP‐1^−/−^* mice, we isolated EPCP from WT *Pax6‐LacZ^+^* donor LGs as described above. Cells were injected into the LG of 6‐ to 8‐month‐old *TSP‐1^−/−^* mice. Evaluation of EPCP engraftment was performed at 20, 40, and 80 days after transplantation.

### Lacrimal Gland X‐Gal Staining

Donor LGs were fixed with 1.25% paraformaldehyde and 1.25% glutaraldehyde solution and processed for 5‐bromo‐4‐chloro‐3‐indolyl β‐D‐galactoside (X‐Gal) staining as described previously 19.

### Histology, Cell Quantification, and Evaluation of Cell Engraftment

#### Analysis of Cell Engraftment in IL‐1α Injured LG

LGs stained with X‐Gal were embedded in paraffin, and 5‐ to 10‐μm sections were costained with Fast Red to visualize the nuclei. Slides were scanned with a Leica scanner (Leica Microsystems, Wetzlar, Germany, http://www.leica-microsystems.com). To determine the efficiency of recipient cell engraftment after LG acute injury with IL‐1α, the number of LacZ^+^ and LacZ^−^ cells were counted on serial LG sections, and engraftment was estimated by quantification of the ratio between LacZ^+^ and LacZ^−^ cells. Cells were counted within the fields of 200 μm^2^ (8 fields per section, 15 sections per lacrimal gland, and 8 LGs per each time point were analyzed}. Three independent experiments were performed.

#### Analysis of Cell Engraftment in TSP‐1^−/−^ LG

To evaluate cell engraftment into “diseased” LGs of the *TSP‐1^−/−^* recipient mice, LGs were isolated at different time points (20, 40, 60, and 80 days) after EPCP transplantation, fixed, and processed for X‐gal staining. Serial paraffin sections (5 μm in thickness) were prepared from recipient and control (vehicle‐injected) LGs and stained with Fast Red to visualize nuclei. Every third section was collected for further analysis. Engraftment was assessed as the percent of LacZ^+^ donor‐derived cells in each injected LG lobe. Cells were counted in 200‐μm^2^ fields, with 8 fields per section and 15 sections per LG. Four LGs were assessed at each time point in each of three independent experiments (i.e., in total 12 LGs [180 sections] were analyzed per time point). Quantification and measurement of inflammatory foci were performed by using a Leica DM4 B microscope and LAS X integrated imaging system.

### Statistical Analysis and Data Presentation

Statistical analyses were performed by using Prism Software (GraphPad, San Diego, CA, http://www.graphpad.com). In bar graphs, data are presented as means ± SD of replicates from a representative experiment or of the normalized data from several experiments. In the latter case, mean fold changes were calculated by first determining the ratio of the test condition over the appropriate control condition for each individual measurement, and then averaging these ratios. The unpaired two‐tailed Student's *t* test and two‐tailed Fisher's exact test were used to determine significance (*p* < .05) in the differences between data sets.

## Results

### Isolation of Putative LG EPCPs by Fluorescence‐Activated Cell Sorting

**Figure 1:**
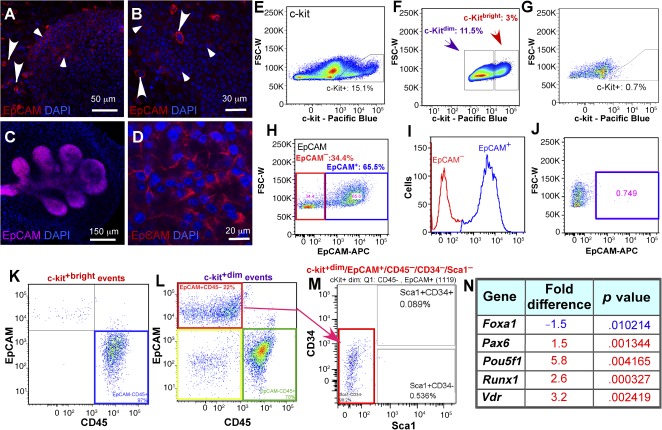
Characterization and isolation of c‐kit‐ and EpCAM‐positive cells. **(A, B):** c‐kit expression (red) in embryonic **(A)** and adult **(B)** lacrimal gland (LG). **(C, D):** EpCam expression (red) in embryonic **(C)** and adult **(D)** LG. Large c‐kit^+^bright cells are seen in the mesenchyme (large arrowheads) and small c‐kit^+^dim cells (small arrowheads) in the epithelium (blue indicates DAPI‐stained nuclei). **(E–G):** Flow cytometric profile of freshly isolated c‐kit^+^ LG cells **(E)**, gating of c‐kit^+^bright and c‐kit^+^dim cells **(F)**, and isotype control antibody staining shows no labeled cells **(G)**. **(H, I):** Gating of LG EpCAM^+^ cells **(H, I**: red square in **H** and red peak in **I** are EpCAM^−^ cells; blue square in **H** and blue peak in **I** are EpCAM^+^ cells). **(J):** Isotype control antibody staining shows no labeled cells (blue square). **(K):** Gating the c‐kit^+^bright cells: all c‐kit^+^bright cells are EpCAM^‐^/CD45^+^ (blue square). **(L):** Gating of c‐kit^+^dim/EpCAM^+^/CD45^‐^ cells. C‐kit^+^dim events: red square, c‐kit^+^dim/EpCAM^+^/CD45^−^ cells, 22%; green square, kit^+^dim/EpCAM^−^/CD45^+^ cells, 70%; yellow square, kit^+^dim/EpCAM^−^/CD45^−^ cells, less than 8%. **(M):** Gating and isolation of epithelial progenitor cell (EPCP) (c‐kit^+^dim/EpCAM^+^/CD45^−^ /CD34^−^/Sca1^−^ red square). **(N):** Putative EPCPs express stem cell markers. Gene expression in sorted EPCPs (c‐kit^+^dim/EpCAM^+^ cells) were compared with isolated c‐kit^−^/EpCAM^+^ epithelial cells using a RT2 First Strand Kit (catalog no. 330401; SABiosciences, Qiagen) and applied to The Mouse Stem Cell Transcription Factors RT^2^ Profiler Array. Only genes changed 1.5‐fold with significantly different expression levels (*p* < .01) are shown. Statistical analysis was performed by using real‐time polymerase chain reaction data from three independent RNA preparations. Abbreviations: DAPI, 4′,6‐diamidino‐2‐phenylindole; EpCAM, epithelial cell adhesion molecule; FSC‐W, forward scatter pulse width.

We selected a panel of potential markers for LG progenitor cells based on existing publications. CD‐117, also known as c‐kit, is the receptor for stem cell factor, a cytokine involved in stimulating a variety of stem cells to cycle. Although c‐kit was originally described on hematopoietic stem cells, this receptor is found on numerous stem cell types 21 22 23 24 25. c‐kit^+^ cells restored function of irradiated salivary glands 26 and regenerated the prostate gland 27. We assessed the distribution of c‐kit^+^ cells by immunostaining of whole LGs (Fig. 1A, 1B) using the c‐kit antibody. At least three types of c‐kit^+^ cells were observed: small and large bright granular cells were scattered throughout the mesenchyme (Fig. 1A, 1B, large arrowheads), and small dim c‐kit^+^ cells were seen in the epithelium (Fig. 1A, 1B small arrowheads).

To distinguish between epithelial and mesenchymal c‐kit^+^ cells, we used EpCAM. EpCAM is reported to mark undifferentiated and differentiated epithelial cells in multiple glandular tissues. Immunostaining of embryonic and adult LGs with the EpCAM antibody (Fig. 1C, 1D) confirmed that EpCAM expression was restricted to the LG epithelial cell lineage.

Similar to our immunostaining results, c‐kit^+^dim and c‐kit^+^bright cells were evident by flow cytometry (Fig. 1E–1G; supplemental online Fig. 2C). The EpCAM antibody detected a single population of EpCAM^+^ cells (Fig. 1H–1J; supplemental online Fig. 2D).The isotype control antibodies showed no staining (Fig. 1G, 1J). We also used antibodies to stem cell antigen 1 (Sca1) (expressed in multiple stem/progenitor cells), CD34 (hematopoietic and mesenchymal progenitor cell antigen), and CD45 (hematopoietic cell marker). In separate experiments, we also tested the antibody to CD31 (platelet endothelial cell adhesion molecule). The last three markers were used to exclude mesenchymal, hematopoietic, and endothelial cells. Once conditions for specific staining with a single antibody were determined ( supplemental online Fig. 2C–2H), we used combinations of antibodies for flow cytometry analysis. All cells in the c‐kit^+^bright populations were EpCAM^−^, but CD45^+^ (Fig. 1K). Thus, c‐kit^+^bright cells have a nonepithelial origin and could represent hematopoietic/endothelial progenitors and possibly mast cells. Analysis of c‐kit^+^dim cells (Fig. 1L) with EpCAM and CD45 antibodies revealed three populations: c‐kit^+^dim/EpCAM^+^/CD45^−^ (22%, or 2.5%–3.0% of total cells; red square), c‐kit^+^dim/EpCAM^−^/CD45^+^ (70% or 8% of total cells; green square), and small c‐kit^+^dim/EpCAM^−^/CD45^−^ (Fig. 1L; <1% of total cells; yellow square). Further analysis of these cells (Fig. 1M) using Sca1 and CD34 antibodies revealed that the only epithelial (EpCAM^+^) cell population was negative for both of these markers (i.e., c‐kit^+^dim/EpCAM^+^/CD45^−^/CD34^−^/Sca1^−^ [2.2%–2.7% of total cells]) (Fig. 1M, red square). This cell population was also CD31‐negative (data not shown). We hypothesized that c‐kit^+^dim/EpCAM^+^/CD45^−^/CD34^−^/Sca1^−^ cells represent a putative LG EPCP population.

### FACS‐Isolated EPCP Cells Express Runx1 and the Pluripotency Factor Oct4

To further characterize EPCPs, we compared sorted EPCPs (c‐kit^+^dim/EpCAM^+^/CD45^−^/CD34^−^/Sca1^−^) with c‐kit^−^ epithelial cells (comprised of partially differentiated and differentiated cells) (c‐kit^−^/EpCAM^+^/CD45^−^) for expression of stem cell transcription factors using the Stem Cell Transcription Factor Array (Qiagen). We found that EPCPs were highly enriched for pluripotency factor octamer‐binding transcription factor 4 (Oct4: 5.8‐fold increase; *p* = .004165), also known as POU5F1 (POU domain, class 5, transcription factor 1). Oct4 is frequently used as a marker for undifferentiated cells and is critically involved in the self‐renewal of epithelial stem cells 28, 29. Oct4 is also one of the reprogramming factors that allows somatic cells to be converted to induced pluripotent stem cells 30, 31. In addition, putative EPCPs also expressed high levels of Runt‐related transcription factor 1 (also known as acute myeloid leukemia 1 protein or core‐binding factor subunit α‐2) (3.6‐fold; *p* = .000327) and vitamin D receptor (Vdr) (3.2‐fold; *p* = .002419) (Fig. 1N). Vitamin D metabolism has been reported to modulate expression of pluripotency genes and cell differentiation 32. Expression of Pax6 was moderately increased in EPCPs relative to epithelial EpCAM^+^/c‐kit^−^ cells (∼1.5‐fold, *p* = .001344), whereas the expression levels of Sox9 and Sox6, previously found to be important during LG growth in embryonic development 19, 33, are equivalent in EPCP and the EpCAM^+^/c‐kit^−^ cells (data not shown). The expression of forkhead box protein A1, which plays a critical role during embryonic development and tissue differentiation 34 35 36, was moderately lower in EPCP than in EpCAM^+^/c‐kit^−^ cells (−1.5‐fold; *p* = .010214). These data give further support to the hypothesis that isolated c‐kit^+^dim/EpCAM^+^/CD45^−^/CD34^−^/Sca1^−^ cells are LG epithelial cell progenitors.

### Establishment of Mouse Epithelial Cell Progenitor Cultures With the Ability to Differentiate Into Secretory Cells

**Figure 2:**
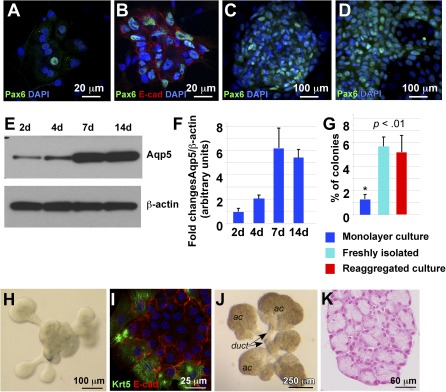
Differentiation of lacrimal gland (LG) epithelial cells in monolayer culture. **(A–D):** One day **(A)**, 1 week **(B, C)**, and 3 weeks **(D)**. **(A–D):** Cultured cells were stained with antibody to Pax6 (green). **(B):** Cells were stained with antibodies to Pax6 (green) and E‐Cadherin (red). Nuclei are stained with DAPI (blue). **(E):** Western blot showing expression of aquaporin‐5 (Aqp5) in fluorescence‐activated cell sorting‐isolated epithelial progenitor cells (EPCPs) cultured for 2, 4, 7, and 14 days. **(F):** Quantification of protein abundance as measured from immunoblot band intensities (e.g., **E**) in three independent experiments. Values represent the optical density of Aqp5 bands normalized to that of β‐actin bands (means of three experiments ± SD). **(G):** Comparison of colony forming abilities (using an in vitro colony forming efficiency [CFE] assay) of EPCPs grown in monolayer, freshly isolated EPCPs, and EPCPs grown in three‐dimensional (3D) reaggregated cultures. The CFE was calculated as the number of colonies at day 8 as a proportion of the number of cells plated in a well. In each experiment, six replicate wells were analyzed per each experimental condition. The final CFE was calculated in three independent experiments (i.e., a total of 18 wells per each condition). *p* value was determined versus control (freshly isolated EPCPs). Asterisk indicates significant differences between monolayer cultures and freshly isolated or reaggregated cultures: *, *p* < .01. **(H):** EPCPs grown in 3D reaggregated culture form buds in 24–48 hours. **(I):** Buds express Krt5 (green) and E‐cadherin (red). **(J):** LG reaggregates cultured for 1 week differentiate into ductal and acinar components. **(K):** Transverse section of one of the acinar components of the 3D reaggregated culture showing acinar structure. Section was stained with Fast red to visualize nuclei. Abbreviations: ac, acinar; DAPI, 4′,6‐diamidino‐2‐phenylindole; duct, ductal; E‐cad, E‐cadherin.

We previously showed that Pax6 is expressed during LG development in proliferating epithelial cells 18. When plated, EPCPs formed small aggregates adherent to the substrate that showed multiple cell divisions. A subset of cells within each primary aggregate expressed Pax6 (Fig. 2A, green). In 5–7 days, the aggregates increased in size substantially, continued to express Pax6 (Fig. 2B, green), and also started to express other epithelial markers, such as E‐cadherin (Fig. 2B, red). In 2 weeks, the proportion of cells expressing Pax6 increased further (Fig. 2C, green), and in 3 weeks, the cells spread throughout the plate (Fig. 2D, green). This increasing proportion of Pax6^+^ cells is consistent with recent findings that in epithelium, Pax6 marks a highly proliferative population of committed transit amplifying progenitors 37 38 39.

Next, we studied whether EPCPs could differentiate in culture. Replacement of growth medium with differentiation medium (medium with low EGF and bFGF content) induced differentiation as shown by Western blot analysis of the specific acinar differentiation marker Aqp5 (Fig. 2E, 2F). We found that EPCPs cultured for 2–4 days had low levels of Aqp5 expression, whereas cells cultured for 7–14 days had relatively elevated Aqp5 expression (Fig. 2E, 2F). These findings suggest that isolated EPCPs contain proliferating progenitor cells that can differentiate into secretory cells.

### Reaggregated Cultures Preserve Colony‐Forming Capability of EPCPs

LG‐derived cell cultures show a decrease in cell proliferation over time, suggesting that the monolayer cultures do not support long‐term progenitor cell survival/expansion. The cells within the intact LG are organized into three‐dimensional (3D) structures 40, 41. This 3D organization is critical for normal lung, cartilage, mammary gland, and many other organ progenitor cell functions 42 43 44. We developed a simple and efficient technique for 3D culture of LG‐derived EPCPs, which allowed enzymatically disaggregated EPCPs to regain cell‐cell contacts by reaggregation ( supplemental online Fig. 1). FACS‐sorted EPCPs were dissociated in 50 µl of growth medium and drawn into a pipette tip, and the tip was sealed with parafilm. Cells were than pelleted, and the reaggregate was inoculated into a 15‐µl drop of laminin‐111 (Trevigen) gel or Matrigel (BD Bioscience) on a polycarbonate filter floating in growth medium. We reasoned that complex cell‐cell and cell‐matrix interactions in combination with growth factors might preserve the progenitor cell properties of EPCPs. We maintained these 3D cultures in parallel with monolayer control cultures for 3 weeks and then dissociated and seeded cells into six‐well plates for in vitro colony‐forming efficiency assays. Freshly isolated EPCPs were used as an additional control. The colonies were counted at day 8, and CFE was calculated as the number of colonies as of day 8 as a proportion of the number of cells plated (Materials and Methods). Both freshly isolated LG cells and the cells obtained from the 3D cultures formed five times more colonies (as a percentage of cells plated) than cells obtained from monolayer cultures (monolayer cultures = 1.09% ± 0.5%; *p* = .010241; freshly isolated cells = 5.58 ± 1.11%, *p* = .001524; 3D cultures = 5.07% ± 0.89%, *p* = .005212) (Fig. 2G). This experiment showed that reaggregated 3D cultures are able to preserve the progenitor properties of EPCPs.

Moreover, we tested whether cellular reaggregates could differentiate into branched structures in differentiation medium supplemented with FGF10. We found that reaggregated EPCP cultures formed primary buds in 48–72 hours (Fig. 2H), and these buds expressed epithelial marker E‐cadherin and stem cell marker Krt5 19 (Fig. 2I). Moreover, in 7–10 days these cultures formed well‐distinguished ductal and acinar components (Fig. 2J, 2K). This finding suggests that FACS‐isolated EPCPs represent a progenitor cell population capable of undergoing branching morphogenesis.

### Putative EPCPs Engraft Into Injured and “Diseased” LGs

**Figure 3:**
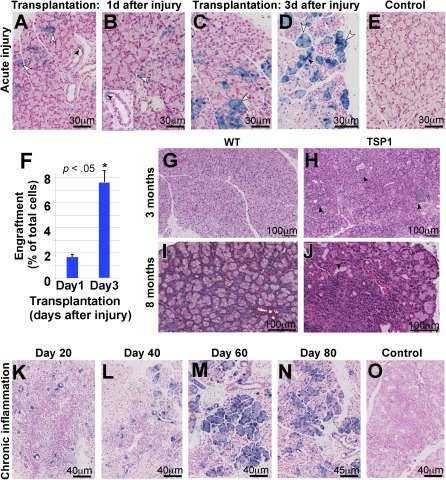
Epithelial progenitor cell (EPCP) transplantation in models of acute and chronic lacrimal gland (LG) inflammation. **(A–D):** Donor EPCPs (LacZ^+^) injected into host LG 1 day **(A, B)** and 3 days **(C, D)** after the interleukin‐1α injury engraft into the recipient's LGs. LG were analyzed 40 days after the transplantation. LacZ^+^ cells are found in acinar (white arrowheads) and ductal compartments (black arrowheads). **(E):** Control (injected with the vehicle) shows no LacZ cells. All sections are costained with Fast Red to visualize nuclei. **(F):** Quantification of EPCP engraftment into recipient's injured LG 40 days after EPCP cell transplantation. Engraftment of cells injected 3 days after the injury is more efficient than engraftment of cells transplanted 1 day after the injury. Engraftment was determined as a percent of the ratio between LacZ^+^ and LacZ^−^ cells. Eight LGs per each time point were analyzed in three independent experiments. Asterisk indicates significant differences in cell engraftment between transplantations performed on days 1 and 3 after LG injury; *, *p* < .05. **(G–J):** *TSP‐1^−/−^* mice progressively develop a severe inflammation in the LG. LG of 3‐month‐old **(G)** and 8‐month‐old **(I)** WT mice show normal acinar structure, whereas the LG of 3‐month‐old *TSP‐1^−/−^* mice show numerous periductal cell infiltrates (**H**, black arrows) and by 8 months *TSP‐1^−/−^* mice develop severe LG inflammation (**J**, black arrows indicate large inflammation foci). **(G, H):** H&E is shown. **(I, J):** Trichrome staining is shown. **(K–O):** Engraftment of LacZ^+^ EPCPs into chronically inflamed LG of 8‐month‐old *TSP‐1^−/−^* mice: analysis of cell engraftment was performed 20 **(K)**, 40 **(L)**, 60 **(M)**, and 80 **(N)** days after cell transplantation. **(O):** Negative control, saline‐injected LG. Abbreviation: WT, wild‐type.

To test the ability of EPCPs to engraft into injured LGs, we performed direct intraglandular transplantation of sorted EPCPs obtained from *Pax6‐LacZ* mice 18, 45 into LGs of nontransgenic recipients (both recipient and donor mouse colonies had the same *C57BL/6* genetic background). Before cell transplantation, the recipient LG was injured by IL‐1α injection 10, and sorted EPCPs were transplanted at 1 day (Fig. 3A, 3B) or 3 days (Fig. 3C, 3D) after the injury. Analysis of cell engraftment was carried out at 40 days after cell transplantation. In both cases (1 and 3 days after the injury), EPCPs injected into the LG were found in the ductal (Fig. 3A, 3B, 3D, black arrows) and acinar epithelial compartment (Fig. 3A–3D, white arrowheads). Control LGs injected with the vehicle did not show any LacZ^+^ cells (Fig. 3E). Interestingly, EPCPs transplanted into uninjured LGs showed no or minimal engraftment (data not shown). Moreover, cells injected 3 days after the injury (at the beginning of the LG regeneration phase) engrafted more efficiently (7.51% ± 0.73%, *p* < .001) than cells injected 1 day after LG injury (LG damage/inflammation phase) (1.43% ± 0.07%; *p* < .001) (Fig. 3F). Thus, putative EPCPs were able to engraft into the LG epithelial compartment, and the efficacy of engraftment depended on the phase of LG regeneration.

Next, we studied whether EPCPs could restore “diseased” LGs of the recently reported and relatively new model of ADDE—the *TSP‐1^−/−^* mouse. Similar to SjS patients, SjS‐specific autoantibodies were detected at elevated levels in the *TSP‐1^−/−^* mice sera. *TSP‐1^−/−^* mice also have elevated levels of interleukins and IFN‐γ, and, importantly, they gradually develop severe inflammation of the LG with infiltrates containing CD4 and CD8 cells, similar to humans with SjS 14, 46, 47. Analysis of Tsp‐1 expression pattern in the LG showed that Tsp‐1 protein is expressed at the apical surface of ductal epithelial cells ( supplemental online Fig. 3A, 3B; white arrows) and in the elongated cells surrounding secretory acini ( supplemental online Fig. 3A, 3B). This pattern of Tsp‐1 expression suggests that, in addition to the ductal cells, Tsp‐1 may be found in the endothelial or myoepithelial cells. Coimmunostaining with the platelet endothelial cell adhesion molecule‐1 (blood vessel marker) antibody showed that Tsp‐1 was not expressed in the small blood vessels ( supplemental online Fig. 3C, 3D, red arrows), but was found in the external layer of cells (most likely pericytes) surrounding large blood vessels ( supplemental online Fig. 3D, white arrowhead). Coimmunostaining with the α‐smooth muscle actin (marker of myoepithelial cells) antibody showed that Tsp‐1 was expressed in all myoepithelial cells ( supplemental online Fig. 3E, 3F, yellow arrows).

**Figure 4:**
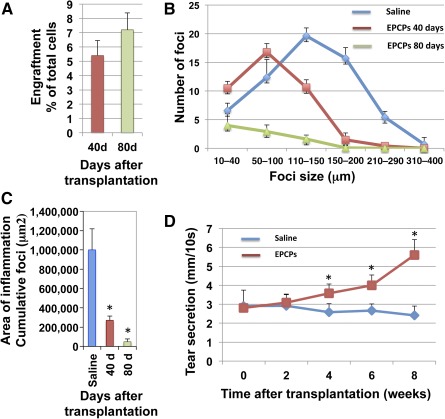
EPCP transplantation improves lacrimal gland (LG) structure and function. **(A):** Analysis of EPCP engraftment into “diseased” LG of 8‐month‐old *TSP‐1^−/−^* mice 40 and 80 days after cell transplantation. A total of 180 sections from 12 EPCP‐injected and 12 control LGs were analyzed per each time point. **(B):** Transplantation of EPCPs decreases inflammation of host (8‐month‐old *TSP‐1^−/−^*) LG with advanced stage aqueous‐deficiency dry eye. The data are mean number of inflammatory foci ± SD from four or five mice. Statistical significance of differences between groups was calculated by using a two‐tailed *t* test for groups with unequal variance and gave the following *p* values: saline‐injected versus EPCP‐injected (60 days after injection), *p* = 1.05658530891 × 10^8^; saline‐injected versus EPCP‐injected (80 days after injection), *p* = 2.53814392935 × 10^14^; EPCP‐injected (60 days after injection) versus EPCP‐injected (80 days after injection), *p* = 7.56001815449 × 10^11^. **(C):** Total area of inflammation (cumulative foci [μm^2^]) in *TSP‐1^−/−^* mouse LG decreases after EPCP transplantation (area of inflammation was calculated at 40 and 80 days after EPCP transplantation in three independent experiments, with four animals per condition in each experiment). In all experiments shown in **A–C**, 180 sections from 12 EPCP‐injected and 180 sections from 12 control saline‐injected LGs were analyzed per each time point. Asterisks indicate significant differences in size of area of inflammation (measured in mm^2^) between saline‐ and EPCP‐injected LGs; *, *p* < .05. **(D):** Animals (8‐month‐old *TSP‐1^−/−^* mice) that received a single injection of EPCPs showed a significant increase in tear production at 4 weeks after transplantation. Tear production was measured in 18 mice per each time point and each condition in three independent experiments (six mice per experiment). The two‐tailed Fisher's exact test was used to identify significant differences between the mean values of experimental and control groups for each time point. The *p* values for each time point were as follows: 0 weeks (*p* = 1), 2 weeks (*p* = 1), 4 weeks (*p* = .06), 6 weeks (*p* = .0002), and 8 weeks (*p* = .002). Asterisks indicate differences in tear production between day 0 and weeks 4, 5, and 6 after EPCP transplantations; *, *p* values are determined in the text above. Abbreviation: EPCPs, epithelial progenitor cells.

Analysis of *TSP‐1^−/−^* mouse LGs showed that inflammation starts at approximately 10–12 weeks as periductal lymphocytic infiltrates *(*Fig. 3, compare 3G and 3H; lymphocytic infiltrates are marked by black arrows), with severe inflammation and formation of large lymphocytic infiltrates evident by 6–8 months of age (Fig. 3; compare Fig. 3G and 3H). We wished to examine whether stem cell treatment could benefit advanced cases of dry eye disease and, in particular, whether donor cells could engraft and differentiate in these conditions. Hence, we used 8‐month‐old *TSP‐1^−/−^* female recipient mice with advanced LG inflammation. The recipient mice were chosen based on tear secretion level: only *TSP‐1^−/−^* mice with significantly decreased (2.5 ± 0.5 mm) aqueous tear production were used (tear production was measured by using phenol‐impregnated cotton threads as described in Materials and Methods). Sorted EPCPs obtained from *Pax6‐LacZ* mice were injected into recipient LGs on one side, whereas the contralateral LG was injected with vehicle as a control. Analysis of cell engraftment and LG morphology was performed at 20, 40, 60, and 80 days after EPCP transplantation (12 recipient *TSP‐1^−/−^* female mice per each time point were used in three independent experiments) (Fig. 3K–3O). At 20 days after transplant, LacZ‐positive EPCPs were mostly incorporated into LG ducts (Fig. 3K); at 40 days after transplant, EPCPs were found in the ducts and differentiating LG acini (Fig. 3L). At 60–80 days after transplant, LacZ‐positive cells were present in the ducts and acini (Fig. 3M, 3N). Comparison of stem cell‐injected and control (saline‐injected) LG morphology showed that density and size of LG inflammatory foci progressively decreased in EPCP cell‐injected LGs, whereas density and size of foci in control LGs remained unchanged (compare Fig. 3K‐3N with 3O). Quantification of cell engraftment at 40 and 80 days after transplant indicated stable long‐term donor engraftment (Fig. 4A). At these stages, we observed a strong reduction in lymphatic cell infiltrations; we also observed the differentiation of LacZ‐positive cells into secretory acini (Figs. 3M, 3N, 4B). We compared numbers and sizes of infiltration foci in LG injected with EPCPs (40 and 80 days after transplant) or with saline using a two‐tailed *t* test. (Fig. 4B), revealing a significant reduction in inflammation in *TSP‐1^−/−^* LGs after EPCP transplantation. We also calculated the statistical significance of the differences in the number of large (>100 μm) and small (<100 μm) foci in experimental (40 and 80 days after EPCP cell transplantation) and control (saline‐injected) LGs using a two‐tailed Fisher's exact test. The numbers of large and small foci in saline‐ and EPCP‐injected (40 days after transplantation) LGs differed, with *p* < .0001; saline‐ and EPCP‐injected (80 days after transplantation) differed with *p* < .0001. Comparison of numbers of foci in the LG of EPCP‐injected mice at 40 days versus 80 days after transplantation showed a significant difference, with *p* < .03. In addition, the cumulative area of inflammation (summed area of infiltration foci, measured in square micrometers) in these two EPCP‐injected groups (40 and 80 days after transplantation) was compared with control saline‐injected LGs. We found that EPCP‐treated LGs had substantial reduction of cumulative area of inflammation (Fig. 4C). These tests indicate significant progressive improvement of the “diseased” LG structure, particularly reduction in inflammatory foci, up to 80 days after EPCP transplantations.

### EPCP Cell Transplantation Improves LG Function

We performed functional assessment of the tear production using phenol‐red‐stained cotton threads (Zone‐Quick; Oasis Medical, Glendora, CA, http://oasismedical.com) as we described previously 14. Estimation of tear production was performed at 0, 2, 4, 6, and 8 weeks after EPCP transplantation (Fig. 4D) and compared with tear production in control saline‐injected LGs. At least six female mice per each time point and each treatment condition were used in each experiment, and the experiment was repeated three times. The two‐tailed Fisher's exact test was used to identify significant differences between the mean values of experimental and control groups for each time point. This test showed significant differences in tear production between EPCP‐ and saline‐injected (control) *TSP‐1^−/−^* mice at 6 and 8 weeks after cell transplantation (Fig. 4D). Comparison of saline‐injected mice at 0‐ and 8‐week time points showed no difference in tear production (*p* = .27), whereas comparison of EPCP‐injected mice at 0‐ and 8‐week time points showed a significant difference (increase) in tear production (*p* = .0002). The results suggest a significant progressive improvement of LG function after EPCP transplantation in an LG disease model.

## Discussion

Our studies suggest that mouse LG contains at least one putative EPCP (c‐kit^+^dim/EpCAM^−^/CD45^−^/CD34^−^/Sca1^−^) population that is able to engraft into the epithelial (acinar and ductal) component of injured/regenerating or “diseased” LG and restore LG structure and function. The c‐kit receptor has been reported to mark epithelial progenitor‐like cells during development of other glands, such as prostate and submandibular gland 27, 48. During submandibular gland morphogenesis, highly proliferative distal buds contain progenitors expressing the c‐kit receptor, which is critical for maintenance and expansion of small mucous granule embryonic progenitor cells 49. Our study shows that the c‐kit receptor is an important marker that identifies adult stem/progenitor cells in LG epithelium. We showed that isolated LG EPCPs possess several important properties of stem/progenitor cells. EPCPs show elevated expression of stem cell markers Runx1, Oct4, Pax6, and Vdr; form colonies; and develop into LG organoids that differentiate into ductal and secretory compartments in 3D cultures. The important role of Pax6 for ocular progenitor cell function was reported in numerous publications 39, 50 51 52 53 54 55 56. It has been reported that Oct4 is critical for self‐renewal and maintenance of multiple stem and progenitor cell types, and Oct4 is downregulated in all differentiated somatic cell types in vitro and in vivo 57, 58. In the corneal epithelium, Oct4^+^ cells were mainly found in limbal and central corneal epithelial basal layers 57. These reports are consistent with our data showing that, in contrast to EPCPs, differentiated epithelial cells of the LG have no or very little Oct4 expression. Enriched expression of the Runx1 transcription factor was also detected in isolated EPCPs. Runx1 is involved in stem cell specification, proliferation, and differentiation in various tissues 59 60 61, and it influences tissue differentiation and homeostasis 60. We previously showed that Runx1 is a marker of the LG epithelial (ductal and acinar) cell lineage 19, 62 during LG development and that Runx1 expression strongly increased during adult LG regeneration 19. We also show that FACS‐isolated EPCPs engraft only into epithelial but not the mesenchymal compartment of the LG, consistent with our hypothesis that EPCPs are epithelial lineage specific stem/progenitor cells.

In the LG acute injury model, transplanted EPCPs engrafted differently depending on the timing of transplantation after injury. The most efficient donor cell engraftment was observed when EPCPs were transplanted 3 days after injury, and they were less efficient at 1 day after injury. Zoukhri et al. previously reported that the most extensive inflammatory response in the IL‐1α‐injured LG was observed on the first and second day after injury, whereas LG remodeling/regeneration was most evident on day three after injury 10. Thus, the state of the host LG is important for successful donor cell engraftment, and the latter is most efficient once inflammation has subsided and regeneration has begun. These findings could inform the development of successful LG repair strategies using stem/progenitor cells, and, in particular, they suggest that suppression of ongoing inflammatory processes may increase the effectiveness of stem cell treatments in chronically “diseased” LGs.

Importantly, we showed for the first time that intraglandular transplantation of EPCPs could restore structure and function of the chronically “diseased” *TSP1^−/−^* LG. We recently showed that expression of important epithelial stem/progenitor cell markers is altered in the LGs of two mouse models of Sjögren's syndrome: nonobese diabetic and *MRL‐lpr/lpr* mice during LG inflammation 63. This finding suggests the importance of the LG state for stem progenitor cell function and the possible role of inflammation in suppressing regeneration. However, our present work shows that EPCP transplantation was effective, even for older mouse recipients with advanced LG inflammation. Transplanted cells were able to not only engraft into LG epithelial compartment, but also to partially restore the structural integrity of the LG acini and improve secretory function. We observed that, even in the areas with low numbers of the engrafted EPCPs, the density of infiltrating cells within the inflammatory foci decreased. We speculate that, as described for some other stem/progenitor cells 64, 65, EPCPs might produce anti‐inflammatory factors that reduce immune cell infiltration. These findings are promising for the development of stem cell therapies to treat LG diseases.

Overall, our study shows that uninjured adult LGs contain epithelial cell progenitors that we define as EPCPs. These EPCP cells could successfully engraft into ductal and acinar compartments of “diseased” LGs and restore acinar structure. We also found that EPCPs could increase tear secretion in *TSP‐1^−/−^* mice with chronic LG dysfunction. Our work provides an important first step toward identifying and characterizing an epithelial‐specific progenitor population able to restore function in the LG. Further analysis of the molecular mechanisms that define and regulate their function within normal and “diseased” LGs could facilitate development of new cell‐based treatments for dry eye and other LG conditions.

## Author Contributions

A.G. and D.A.V.: collection and/or assembly of data, data analysis and interpretation, final approval of manuscript; M.Y. and S.T.: collection and/or assembly of data, final approval of manuscript; R.M.: data analysis and interpretation, manuscript writing, final approval of manuscript; D.A.D.: conception and design, data analysis and interpretation, final approval of manuscript; H.P.M.: conception and design, financial support, data analysis and interpretation, manuscript writing, final approval of manuscript.

## Disclosure of Potential Conflicts of Interest

The authors indicated no potential conflicts of interest.

## Supporting information

Supporting Information
