# Supplementary material for: Lacrimal Gland Repair Using Progenitor Cells
Source: Stem Cells Transl Med. 2016 Aug 15;6(1):88–98. doi: 10.5966/sctm.2016-0191 (PMC5442743; doi:10.5966/sctm.2016-0191)
Supplement: Supplementary file 1 — Supporting Information [file SCT3-6-088-s001.pdf]

Supplemental Figures - Gromova et al.

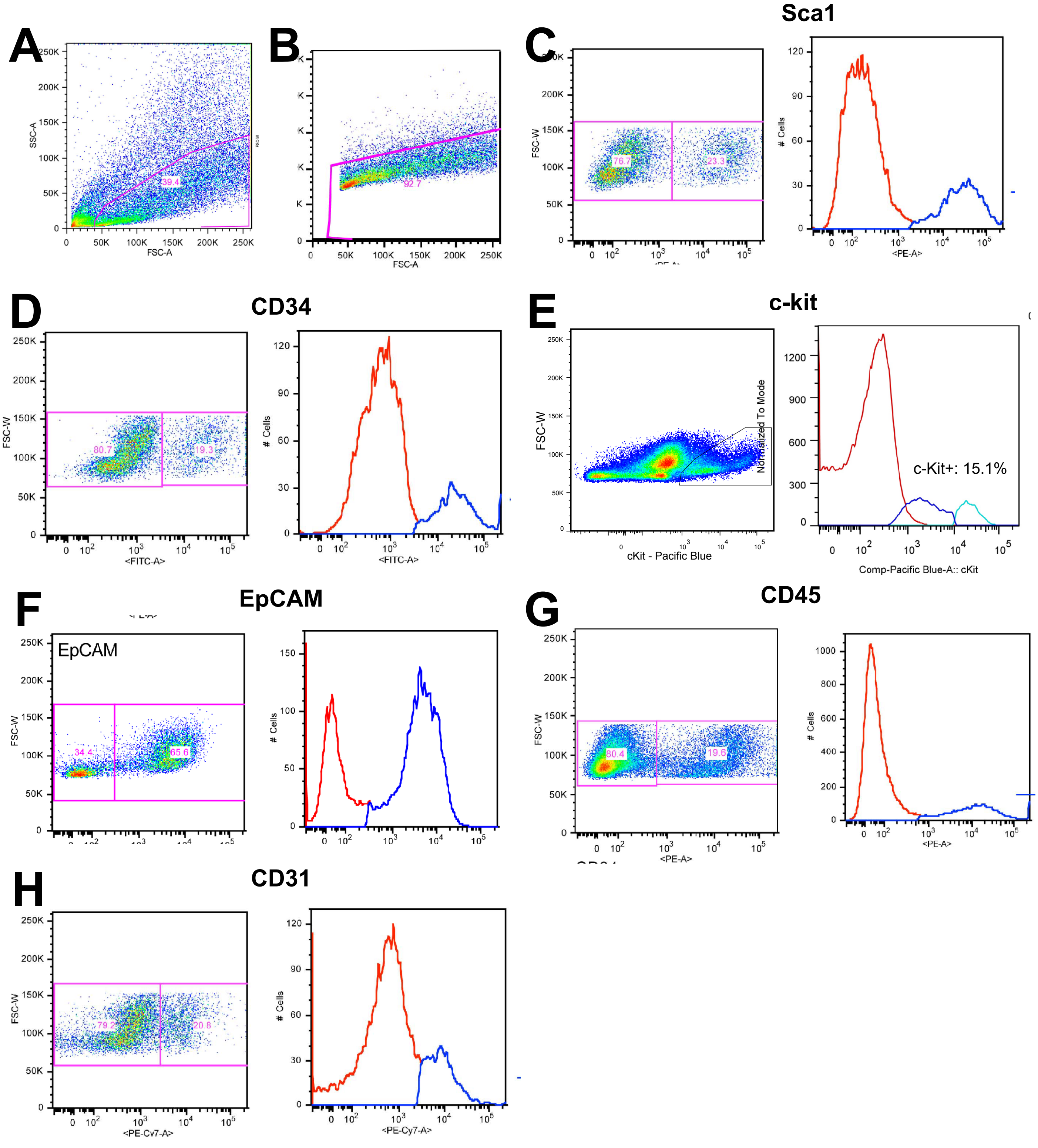

Figure S1. Analysis of LG cells by flow cytometry (A) Identification of the main live cell population. (B) Example of doublet exclusion. (C-H) gating to isolate: (C) Sca1+/Sca1-; (D) CD34+/CD34-; (E) c-ki+/c-kit-; (F) EpCAM+/EpCAM-; (G) CD45+/CD45- (H) CD31+/CD31- cells. For each marker, red lines in the histograms show negatively-stained cells and blue lines show positively-stained cells.

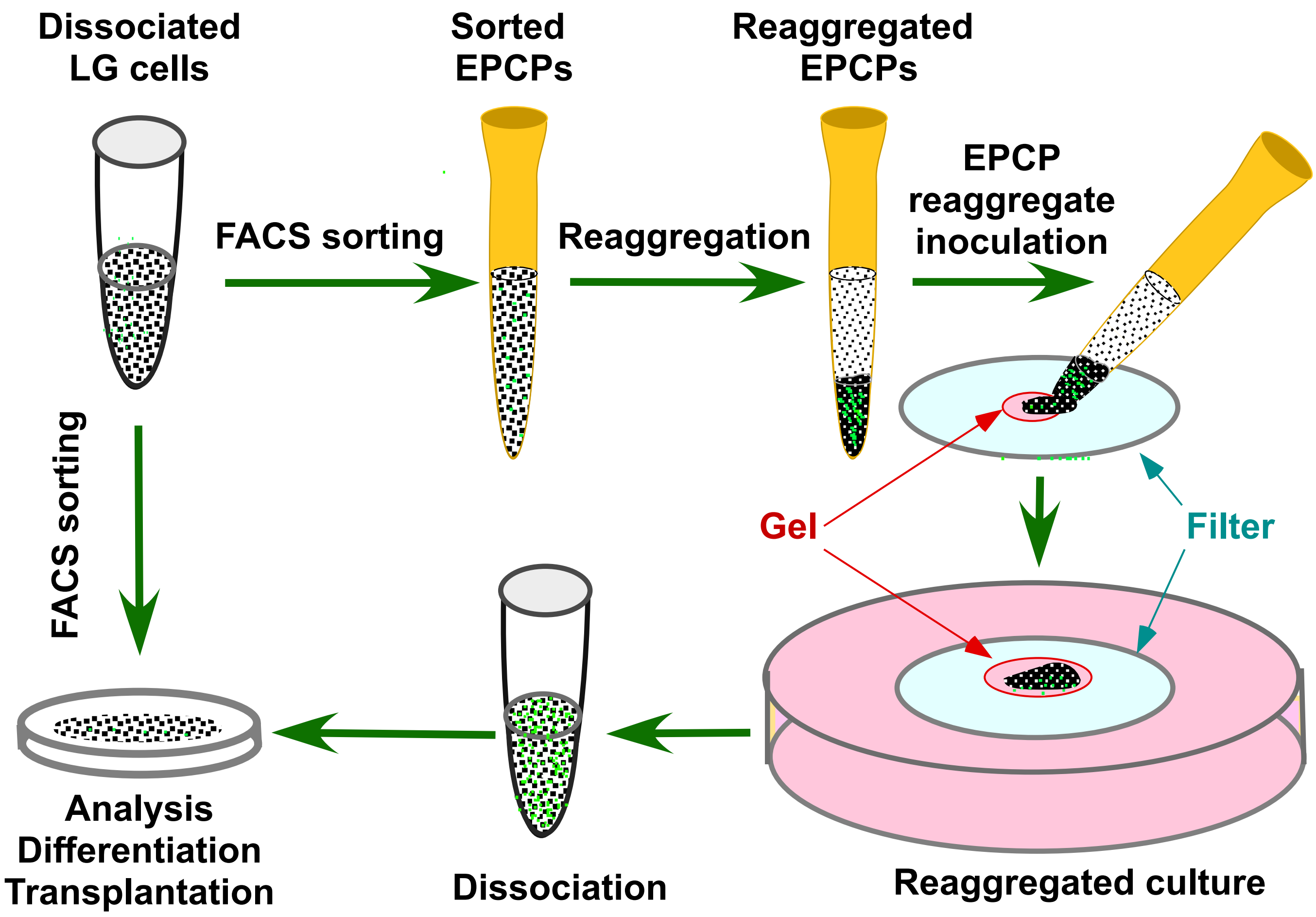

Figure S2. Preparation of EPCP re-aggregated 3D cultures. FACS isolated EPCP's were counted and dissociated in culture medium. 1x105-1.5x106 cells in 50 µl of medium were drawn into a 100µl pipette tip and the tip was sealed with the ethanol washed parafilm. Cells were than pelleted at 1000 g for 10 min and the cell plug/re-aggregate then was inoculated into a 15 µl drop of laminin-111 (Trevigen) gel or matrigel (BD) sitting on a polycarbonate filter and floating in serum-free medium.

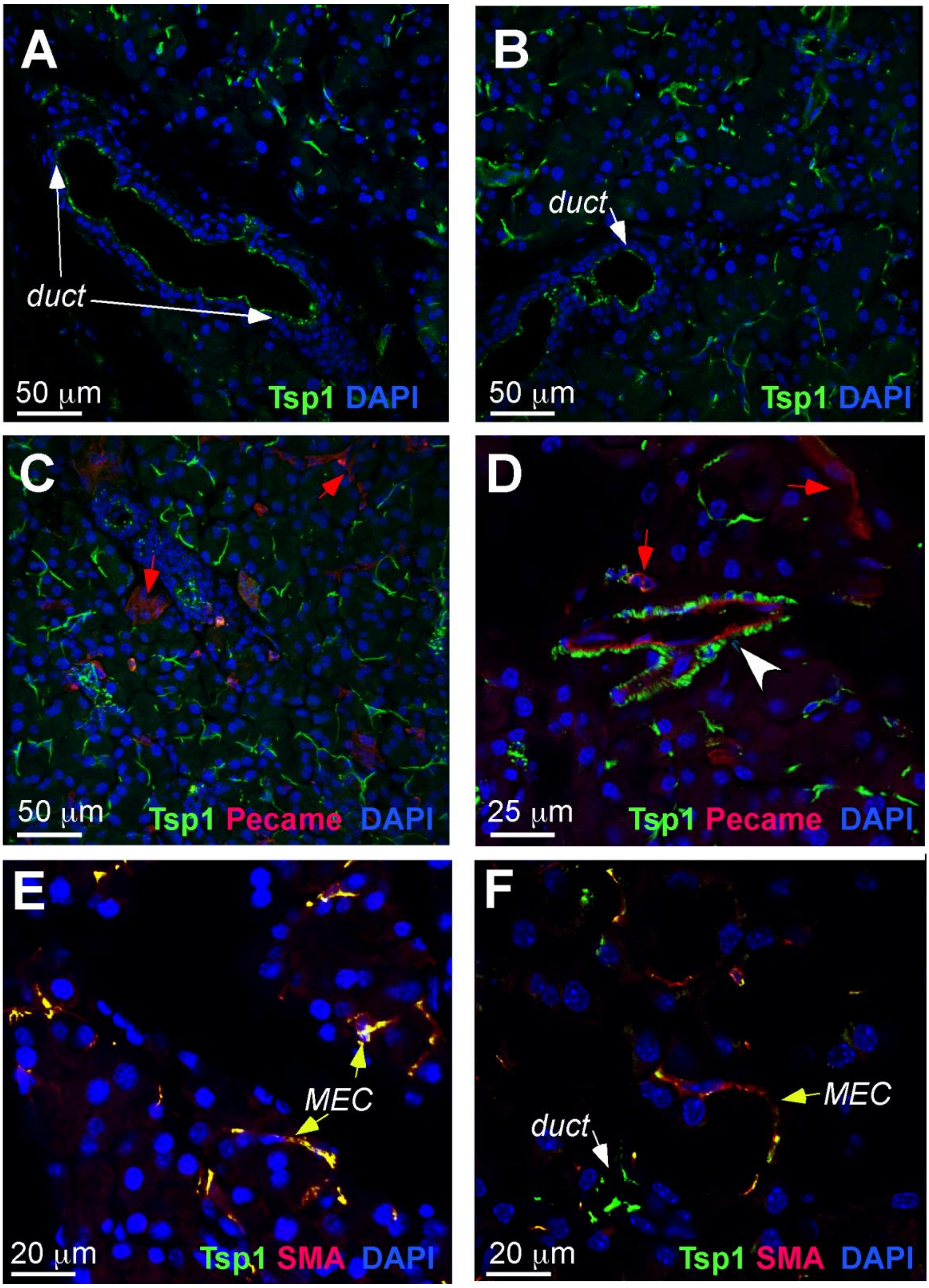

Figure S3. Tsp-1 expression pattern in the LG. (A-B) In the normal LG, Tsp-1 protein (green) was found at the apical surface of ductal epithelial cells (duct: white arrows) and in the elongated cells surrounding secretory acini. (C, D) Co-immunostaining with the Platelet endothelial cell adhesion molecule-1 (Pecam-1: blood vessel marker (red)) antibody showed that Tsp-1 protein (green) was

not expressed in the LG small blood vessel (red arrows). However Tsp-1 protein was found in the cells (most likely pericytes) on the external surface of large blood vessels (D, large white arrowhead). (E, F) Co-immunostaining with the smooth muscle actin (SMA (red); marker of myoepithelial cells) antibody showed that Tsp-1 (green) was expressed in all myoepithelial cells (MEC, yellow arrows).
